# Managing the TME to improve the efficacy of cancer therapy

**DOI:** 10.3389/fimmu.2022.954992

**Published:** 2022-10-20

**Authors:** Maria Teresa Bilotta, Antonella Antignani, David J. Fitzgerald

**Affiliations:** Laboratory of Molecular Biology, Center for Cancer Research, National Cancer Institute, National Institutes of Health, Bethesda, MD, United States

**Keywords:** anti-cancer therapy, immunosuppression, immunotherapy combined therapy, TME (tumor microenvironment), Hodgkin (cHL), GBM - glioblastoma multiforme, PDAC - pancreatic ductal adenocarcinoma

## Abstract

The tumor microenvironment (TME) influences tumor growth, metastatic spread and response to treatment. Often immunosuppression, mediated by the TME, impairs a beneficial response. The complexity of the tumor composition challenges our abilities to design new and more effective therapies. Going forward we will need to ‘manage’ the content and or functionality of the TME to improve treatment outcomes. Currently, several different kinds of treatments are available to patients with cancer: there are the traditional approaches of chemotherapy, radiation and surgery; there are targeted agents that inhibit kinases associated with oncogenic pathways; there are monoclonal antibodies that target surface antigens often delivering toxic payloads or cells and finally there are antibodies and biologics that seek to overcome the immunosuppression caused by elements within the TME. How each of these therapies interact with the TME is currently under intense and widespread investigation. In this review we describe how the TME and its immunosuppressive components can influence both tumor progression and response to treatment focusing on three particular tumor types, classic Hodgkin Lymphoma (cHL), Pancreatic Ductal Adenocarcinoma (PDAC) and Glioblastoma Multiforme (GBM). And, finally, we offer five approaches to manipulate or manage the TME to improve outcomes for cancer patients.

## Introduction

**Figure 1:**
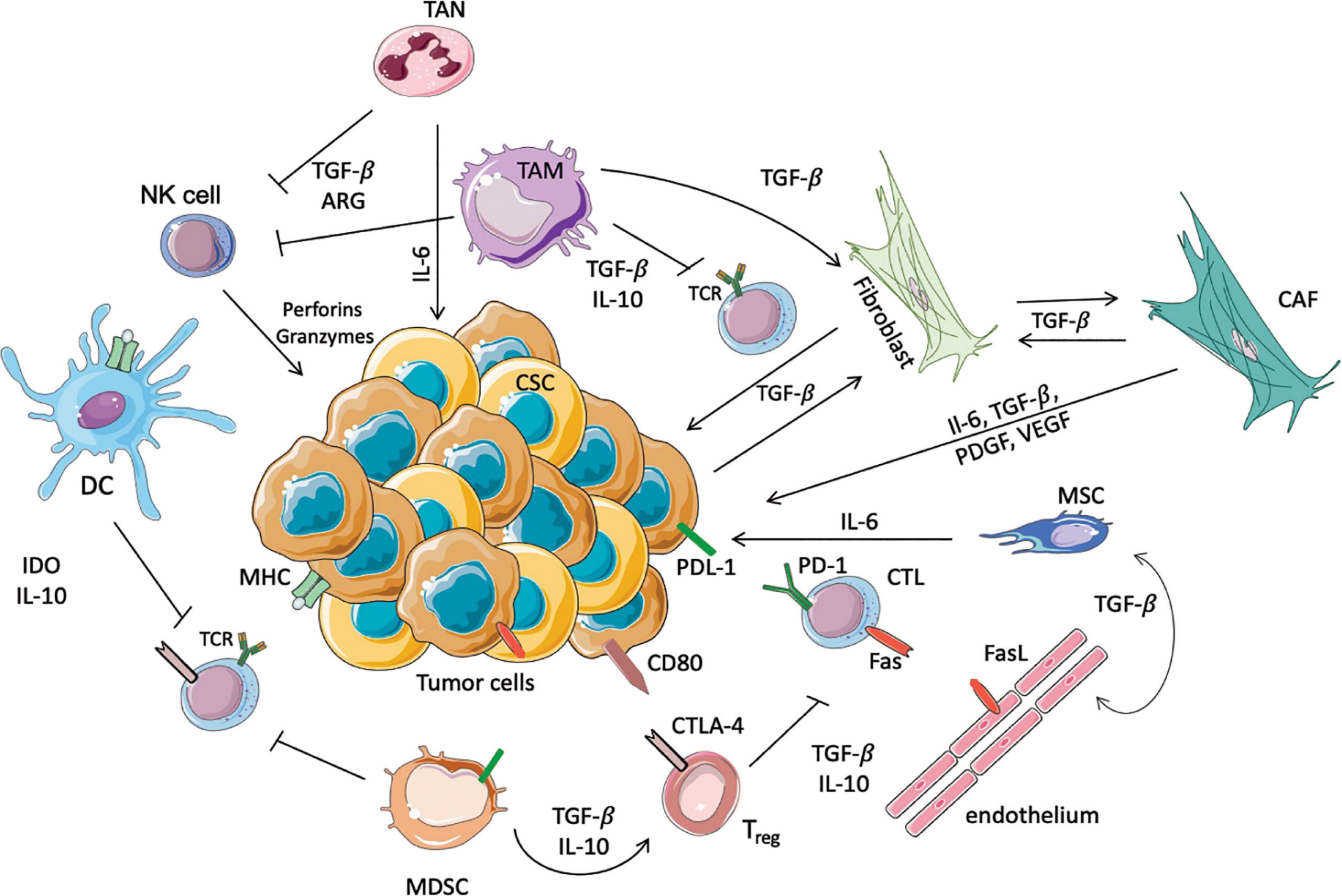
Schematic representation of immunosuppressive cells in the TME. In this scheme the major immune cells involved in the anti-tumor or pro-tumor response are highlighted. TANs and TAMs secrete TGF-β, Il-10 and ARG-I, which inhibit the cytotoxic activity of NK cells and T cells. Moreover, TAMs promote the conversion of normal fibroblasts into CAFs, which in turn promote the proliferation of tumor cells. In this immunosuppressive microenvironment DCs, through the secretion of IL-10 and overexpression of IDO, prevent the activation of T cells, avoiding the recognition of the tumoral antigens expressed on MHC. Another immunosuppressive population is represented by MDSCs that inhibit the activation of T cells, and furthermore allow the activation of Tregs, in particular increasing the expression of CTLA4 on the surface of Treg themselves. Moreover, Tregs inhibit the cytotoxic functionality of the CTLs, where PD-1 and Fas are increased to inhibit the anti-tumoral response. Finally, the endothelium contributes to immunosuppression, since these cells express FasL.

As tumors begin (tumorigenesis) they send out signals that recruit cells as an initial response to emerging disease. If the early influx of immune cells succeeds with surveillance and elimination, malignant cells will be destroyed and a crisis averted. However, if the signals are insufficient or the response inadequate, a tumor emerges, starts to grow locally and eventually can spread to distant sites. Along with the emerging tumor comes a collection of soluble factors, promoting the influx of non-malignant cells, blood vessels and stroma, which together becomes the TME (**Figure 1**). As the tumor progresses, the surrounding TME also changes: these changes are not uniform with respect to structure nor static with respect to time. The TME is undeniably complex and studying it is a major challenge (1). But techniques are improving all the time where notable advances such as intravital microscopy (2, 3), single cell sequencing (4) and cell-location techniques borrowed from astronomy can inform us about the complicated workings of the TME (5). Once better informed, we can expect new interventions that improve therapeutic responses.

The TME frequently contributes to a poor anti-tumor poor response. For the past twenty years, Hanahan and Weinberg have been describing the ‘hallmarks of cancer’ that lead to the neoplastic state (6, 7). In the most recent update, Hanahan now list ten ‘hallmarks of cancer’ that include ‘avoiding immune destruction’ and ‘tumor-promoting inflammation’ (8). Also, mentioned in direct connection with these two elements is the overarching role of the TME (8). Notably, the TME is associated with immune suppression. Unfortunately, suppression comes in many flavors and often defies efforts to reinstate a curative response. Throughout this review, we will highlight problematic features of the TME and suggest ways to ‘manage’ various elements of tumor biology. It will become quickly apparent that almost any treatment that alters the ‘natural’ behavior of the TME comes with a price. For example, adding a checkpoint inhibitor can overcome suppression but risks autoimmunity (9). Also, we know that checkpoint inhibitors perform best against malignancies with a high tumor mutational burden, but (artificially) increasing this burden could cause secondary cancers (see below). Likewise, eliminating myeloid cells has a downside such as allowing the establishment of serious infections (10). Part of the issue is the nature of the target cell population. It is difficult to eliminate the ‘problematic’ normal cells located within the TME without effecting the same populations systemically. For instance, it might be possible to use antibodies to CD25 with the goal of eliminating regulatory T cells (Tregs) (see below for more on this) but this strategy risks reducing the number of activated T cells that could play a pivotal role in the killing of malignant cells. Because of these confounding issues, our theme here is to ‘manage’ the TME for maximum benefit and minimum risk.

Along with updated techniques to characterize the TME, there are new treatment options including small molecular weight drugs, antibody-based agents and transduced lymphocytes, each employed with the goal of improving outcomes for patients with cancer. As preclinical models are assessed and novel strategies tested in the clinic, we expect rules will be established that match ‘treatments to outcomes’ based on both the tumor type and the composition of the TME. In fact, some rules are emerging already. These include malignant cells harboring many mutations generally respond better to checkpoint inhibitors than tumors with a small number of mutations. An off-shoot of this is the situation where mismatch repair pathways are defective, producing additional mutations and leading to even better responses to checkpoint inhibitors (11). This suggests further a strategy whereby certain DNA damaging agents or inhibitors of DNA repair might be employed to artificially generate more mutations leading to an improved response to checkpoint inhibitors. Of course, this approach is not without risks, since introducing additional mutations is unlikely to be confined to malignant cells and there will be a risk of new cancers emerging. Such calculations will be part of the ‘management’ strategy as clinicians evaluate individual patients as they present with distinct cancers and an accompanying TME. We know that some ‘cold’ tumors do not respond to checkpoint inhibitors simply because there are few or no T cells close to the malignant cells. Apparently, there are gatekeeper strategies that keep T cells out of the TME (12, 13). Overcoming these exclusionary mechanisms could be useful in converting cold tumors to hot ones (see below). Again, managing T cell traffic without provoking autoimmunity will be important.

## Composition of the TME

Before we discuss the ‘management’ of the TME, we need to understand its composition (**Figure 1**). Besides the malignant cell, tumors are populated by normal cells of various kinds. These include blood vessels, fibroblasts and immune cells of myeloid or lymphoid origin. The pathway of attraction into the tumor is usually associated with the secretion of soluble signaling molecules such as chemokines or cytokines secreted by cancer cells, immune cells, fibroblasts and mesenchymal stem cells. This secretion allows cross-talk within the TME. Soluble factors not only attract immune cells but can reprogram their function. Is this fashion cells with the potential to fight tumor growth are altered and become part of the suppressive landscape (14). The immunosuppressive and inflammatory status of the TME is characterized by cytokines and chemokines that orchestrate the immune infiltration and the immune response. Upon the recognition of tumor antigens, myeloid cells, especially dendritic cells (DCs) and macrophages produce inflammatory cytokines such as type-1 IFNs, IL-12, IL-15, IL-18, IL-21 involved in the natural killer (NK) cell tumor-killing response (15, 16). The CCR2 inflammatory monocytes are recruited in the tumor by CCL2 (17), and the macrophages are attracted by cancer-associated fybroblasts (CAFs) through SDF-1/CXCL12 expression, indeed SDF-1 magnifies the polarization of the macrophages into tumor- associated macrophages (TAMs) (producing high levels of IL-10). IL-10, and similar anti-inflammatory cytokines and chemokines, cause immune suppression by inhibiting T cells and NK cells. Further, chemokines CCL5, CCL20 and CCL22 recruit Tregs and activate their inhibitory actions *via* production of IL-10 and TGF-β1 (18). Tumor growth can also fundamentally alter myelopoiesis in the bone marrow (BM), leading to the generation of myeloid derived suppressor cells (MDSCs). Cytokines GM-CSF, G-CSF and IL-6 produce MDSCs from precursors, apparently reprogramming the BM and altering the composition of circulating myeloid cells (19, 20). MDSCs can also differentiate into TAMs, which are able to directly suppress CD8 cells *via* nitric oxide synthase-2 (NOS-2) and arginase (ARG-1) secretion (21). The TME is characterized not only by immunosuppressive environment but also by sustained inflammation. This inflammatory status attracts neutrophils in an IL-8-dependent fashion in the TME. Once in the TME, TANs sustain inflammation by releasing nitric oxide (NO) and reactive oxygen species (ROS). TANs also induce T cell apoptosis releasing TNF-α, inhibit T cell proliferation secreting ARG-1 (through the modulation of PD-1/PD-L1 signaling) and are involved in the recruitment of Tregs that further induce an immunosuppressive state by producing CCL17 (22).

This cytokine-mediated recruitment of various suppressive cells into the TME rapidly becomes a barrier to therapy as the accumulation of normal cells can occupy a large fraction of the tumor mass. And when these normal cells promote tumor growth rather than suppress it, the situation is problematic. In fact, the ratio of non-malignant to malignant cells is a fascinating aspect of tumor biology (23) (not a good ref try Mikkilineni et al. Semin Oncol 2017). Included in this perspective is an emerging concept of the immunoscore, which attempts to predict favorable responses based on infiltrating T-cell density (24). Unfavorable responses can also be predicted, especially when the presence of other types of immune cells (e.g. MDSCs) are detected (25). Notoriously, in a number of specific cancers, non-malignant cells can far outnumber the malignant ones. This is especially true for instance in cHL and PDAC. The history of cHL reveals an earlier time when it wasn’t certain that this lymphoma was even a malignancy – there were such few malignant cells. We now know that the Reed-Sternberg (known also as the Hodgkin cell) cell can occupy 10% or less of the total population of the TME. Thus, any effective treatment must negotiate with an ‘army’ of normal cells to eliminate the few malignant ones (26). Likewise, with PDAC, the TME is so ‘dense’ that many treatments fail to penetrate to the malignant cells. In PDAC, this problem has been addressed by testing treatments that disrupt the architecture of the TME (27). So far, no benefit has been achieved with this approach, perhaps because loosening the TME might also promote metastatic spread (28). Again, we are faced with the complexity of unintended consequences and our limited understanding of tumor biology.

Various normal cell types that are found in the TME, include: lymphocytes, myeloid cells, macrophages (both resident and newly arrived from monocytes), neutrophils, fibroblasts and the cellular elements of blood vessel formation (**Figure 1**). As mentioned, together these normal cells often contribute to a suppressive phenotype. Not only do normal pathways of tumor elimination fail to function, but the TME can also mediate resistance to drug or antibody-based treatments. For example, the interaction between the tumoral cells and the extracellular matrix (ECM) through the binding with its components (collagen, fibronectin, and laminin) is responsible for the cell adhesion-mediated drug resistance (CAM-DR), that mediates the resistance to radiotherapy and antibody-based receptor tyrosine kinase (RTK) inhibition (29, 30).

Tumor-associated immune cells ‘should’ play key roles in surveillance which is centered on tumor recognition and elimination. Important cells involved in this process include NK cells, DCs and cytotoxic T lymphocytes (CTLs). These cells are either involved in the recognition or the killing of cancer cells, or both. NK cells are part of the innate immune system and recognize molecules on cancer cells that are rarely expressed by normal cells. The role of DCs is to ingest and process tumor antigens and then present these as peptides in complex with MHC-I (type 1 major histocompatibility complex) molecules to CTLs (**Figure 1**). CTLs then bind and kill tumor cells *via* the release of toxic granules into the cytosol of malignant cells. To prevent an ‘over response’ by CTLs leading to autoimmunity, there are regulatory responses guided by checkpoint molecules and by immunosuppressive cells, such as the Tregs. Further, various myeloid populations that include TAMs and tumor-associated neutrophils (TANs), dampen the immune response (31). The growth of a tumor mass is direct evidence of failure by one or more elements tasked with the elimination of malignant cells.

The connection between the infiltration of leukocytes into tumor lesions and cancer was described in 1863 by Rudolf Virchow, who was the first to suggest that chronic inflammation is relevant to neoplastic progression (32). The myeloid cells of the TME influence not only the proliferation of cancer cells *in situ*, but also their metastatic potential in three ways. 1) The myeloid cells (e.g. TAMs or TANs) promote tumor escape from the primary tumor, and the invasive phenotype is induced by TANs and TAMs, due to the upregulation of beta-catenin expression and downregulation of E-cadherin expression. TANs are also responsible for the degradation of the ECM through proteases and cathepsin secretion. Moreover, the cancer cells use the neutrophils as carriers to enter circulation. Also TAMs enhance the intravasation of the tumor cells (via EGF/CSF1 paracrine loop) (33). 2) Myeloid cells can protect tumor cells in the circulation through the Neutrophil-derived Extracellular Traps (NETs). These are DNA fibers that include histone and cytoplasmic granule proteins. Within NETs, cancer cells can create clusters with neutrophils and release ADP, thrombin and proteinases to stimulate platelet–tumor cell aggregation. These cluster are created also by the binding of tumor cells to L-selectin on neutrophils and to P-selectin on platelets (33, 34). 3) Myeloid cells promote tumor cell extravasation into metastatic sites, that is driven by the expression of vascular cell adhesion protein-1 (VCAM-1) and vascular adhesion protein (VAP) on the endothelium and the release of CCL2 from tumor cells, that further recruit myeloid cells (33, 35).

Moreover, the composition of the TME which is likely determined by the type of cancer, is not static and changes over time. In some tumors immune infiltrates finally outnumber the malignant cells. Tumors also have variable numbers of stromal and endothelial cells, depending on the location of the tissue and the origin of the malignant cells. In solid tumors, the most abundant immune infiltrating cells are TAMs (36). TAMs originate both from the local proliferation of resident macrophages and the infiltration of monocyte derived macrophages (1). While macrophages can exert either a protumor or antitumor phenotype, it is generally understood that increased numbers of TAMs correlate with a poor prognosis and resistance to therapy (37). Because of this, therapies have been designed to reduce the number of TAMs. To date these have not been overly effective in human trials, despite preclinical data describing beneficial outcomes from anti-macrophage therapies (38). Another approach is to switch the TAMs phenotype from protumor to antitumor, mostly through changes in local cytokine levels *via* the use of neutralizing monoclonal antibodies (39) or small molecular weight inhibitors (40). Antibodies that might promote better responses *via* reduced numbers of TAMs include anti-CCL2/anti-CCR2, anti-CSF1R and anti-IL6. And drug treatments include small molecular weight inhibitors of JAK, BTK and PI3K enzymes (40–43). Each of these treatments seeks to alter the TME in a fashion that promotes antitumor responses either alone or in combination with other agents including checkpoint inhibitors (see below).

There is also the special situation found in some hematological cancers, where the malignant cells are themselves immune cells. In these cancers the absence of ECM and the seeding of secondary lymphoid organs (by malignant cells), impairs the anti-cancer immune response. In the secondary organs, indeed, the tumor cells share the same developmental niche with normal immune cells and this event leads to immune paralysis (e.g. in myeloma and chronic lymphocytic leukemia this lead to a reduction in normal B/plasma cells and consequently hypogammaglobulinemia). Some hematological tumor cells can easily cross-talk with normal immune cells in the secondary lymphoid organs (e.g. follicles), where the cancer cells interact with dendritic cells or T-follicular helper and this interactions may also impact immune therapies, such as anti-PD1 immune checkpoint blockade, since PD1 is highly expressed on T- follicular cells (44). Even between solid tumors the TME can change compositions, for example there are “hot” tumors where the immune infiltrate is more abundant and the tumor triggers a strong immune response (e.g. cancers of the bladder, head and neck, kidney, and liver cancers, melanoma, and non-small cell lung cancer), and “cold” tumors (e.g. breast, ovary, prostate, pancreas, cancer and glioblastoma) that are surrounded by cells that suppress the immune response and in some instances actually exclude lymphocytes from close contact with malignant cells. In summary, the composition of the TME influences tumor differentiation, dissemination, and immune evasion.

## Tumors with extraordinary TMEs. PDAC, GBM and cHL

**Figure 2:**
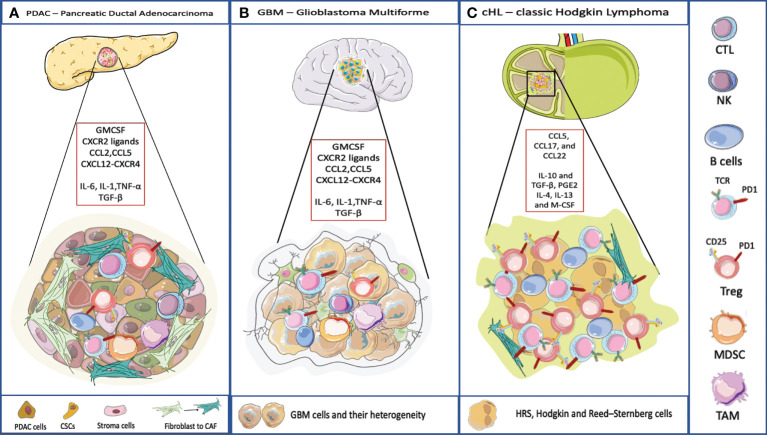
TME and its composition in three different cancer model. **(A)** PDAC TME is characterized by a dense stroma, CAFs, and immune cells populations that crosstalk to the subpopulations of neoplastic cells that include cancer stem cells (CSCs). Pancreatic cancer cells typically express one or more mutated oncogenes or tumor suppressor genes *(*e.g. *KRAS*, *TP53*, *CDKN2A*, and *SMAD4*) that can interact with the TME, regulating stromal cells and the ECM in direct and indirect ways. The TME has highly diverse cellular and extracellular components. The CAFs are responsible for the secretion of extracellular matrix and soluble molecules which create a TME that favors tumor growth and resistance to the therapy. The TME is highly immunosuppressive, with the infiltration of TAMs, MDSC and Treg. The accumulation of CD4+ T cells and B cells contribute to carcinogenesis too. The soluble molecules released by PDAC cells or immune cells recreate an immunosuppressive microenvironment. IL-6, IL-1, TNF-α favor the polarization of macrophages into TAMs and they promote MDSC function, through the secretion of ECM. TGF-β is released by cancer cells and by immunosuppressive cells to favor a pro-tumoral environment. Here are represented some chemokines responsible for the recruitment of immune cells into the tumor, and involved in the redirection of the T cells, for example CCL5 attracts Tregs, CXCL12-CXCR4 restricts lymphocyte migration and keeps CTLs outside the tumor. **(B)** The TME surrounding malignant GBM is very complex. Indeed, the interaction between the immune system and the brain interior is problematic because of the presence of the blood-brain barrier. Even though, also in GBM the TME plays an important role. The GBM cells are heterogeneic they present antigen to CD4 and CD8 T cells that usually are dysfunctional and exhausted. Few B cells infiltrate in the brain. Moreover, GBM is characterized by an immunosuppressive and inflammatory microenvironment composed of cytokines (IL6, IL1-β, TGF-β, IL-10 and prostaglandin E2), chemokines, and regulatory immune-suppressive cells (Treg, TAMs, and MDSCs). Leukocyte recruitment to the tumor site is mediated by inflammatory chemokines from the CXC subfamily and CC group which attract leukocytes within tumor and exert pro- or anti-tumoral effects. CXCL8 is one of the inflammatory chemokines and the axis CXCL8-CXCR1/2 axis belongs to the most important and the best recognized regulatory factors in the development of CNS tumors. Moreover, CXCL8, CCL2, IL-6, contribute to MDSC generation (45). **(C)** cHL is characterized by a peculiar TME, most of the tumor is represented by dysfunctional T lymphocytes and by immunosuppressive cellular and acellular components. IL-10 and TGF-β, produced by RS, allow the accumulation of Tregs involved in the suppression of the activity of effector cells. IL-10 is a potent immune suppressive cytokine. Levels of IL-10 are elevated in up to a half of cHL patients and are correlated to disease aggressiveness and poor response to therapy. TGF-β production contributes to depress T-cell proliferation, cytokine release, and cytolytic activity. The soluble factor, prostaglandin E2 (PGE2), interferes with T cell receptor signaling, and decreases the cytotoxic response. IL-4, IL-13 and M-CSF attract myeloid cells and polarize macrophages into MDSC and TAMs (46).

With the exception of circulating leukemia cells, most tumors have a TME. Here we focus on three particular malignancies where each one has a signature TME. We discuss cHL, PDAC and GBM (**Figure 2**) as models for how the TME can subvert the processes of surveillance and elimination of malignant cells.

### PDAC

PDAC has a dense layer of cancer-associated fibroblasts (CAFs) surrounding malignant cells. CAFs participate in active cross-talk with cancer cells within the tumour microenvironment (47) and contribute to drug resistance (47). CAFs are further classified into myofibroblastic and inflammatory subsets where tumor-promoting inflammatory CAFs are driven by IL-1 and JAK/STAT signaling (48). This kind of report suggests a role in managing the TME *via* antagonists of IL-1 and/or the JAK/STAT pathway. Further in PDAC, MHC-1 surface expression is frequently downregulated, possibly by mechanisms associated with autophagy (49, 50). Finally, PDAC is a classic cold tumor where T-cells are actively excluded. Recent advances from the Fearon Lab suggest that the exclusion zone is dictated by the presence of the heterodimer between Keratin 19 and CXCL12 (12). The dimer being constructed by the action of transglutaminase to form a filamentous network on the outside of malignant cells. How might this work? One hypothesis suggests that CXCL12 binds to CXCR4 on T-cells and thereby stops their migration ability. This might be the equivalent of stopping the tendency of lymphocytes to roll and stop as part of their surveillance. In support of this are data with the compound, AMD3100 a CXCR4 inhibitor that allows greater penetration of T cells into cold tumors – such as PDAC. CXCR4 plays an important role in the retention of stem cells in bone marrow. The addition of AMD3100 allowed for the release of these cells – and by analogy, the addition of AMD3100 to individuals with cold tumors could release them from the CXCL12 block at the periphery of tumors allowing their infiltration deeper in the tumor. Once there, immune checkpoint blockade (ICB), could be useful in overcoming immune suppression.

### GBM

GBM, a tumor of glial cells, is the most aggressive of the malignant gliomas (51). Standard therapy involves surgery and radiation followed by maintenance temozolomide (TMZ) treatment. The TME of GBM is extensive and patients do not respond well to ICB, in part because the tumor has a low mutational burden and in part because the TME is suppressive of T cell functionality. Malignant cells are surrounded by MDSCs, TAMs, TANs and GADCs. Thus, for ICB to be a good option for patients, the suppressive nature of the TME needs to be overcome and/or the mutational burden needs to be increased. In addition, the blood brain barrier acts to restrict the penetration of large molecules such as antibodies into the brain. Generally, tumors are either cold (no T cells), exhausted (T cells are present but not functional) or there are too few targets (low mutational burden) to generate cytotoxic T cell responses. Because TMZ is an alkylating agent (and damages DNA) there may be a way forward, if TMZ is combined with inhibitors of DNA repair, allowing mutations to accumulate, followed finally by treatment with ICB agents (52). This strategy of course risks the extension of the treatment timeline where growing tumors cause irreparable damage to the functional brain.

### cHL

The cHL was first described more than a 100 years ago but was not immediately recognized as a malignancy due to the presence of so many inflammatory cells. Investigators considered the lesions to be probable foci of infection rather than cancer. We now know the presence of the Reed-Sternberg (RS) cancer cell can be present in fewer than 10% of the total cells in a cHL lesion. The TME of cHL is characterized by the presence of several types of lymphocytes, eosinophils and macrophages. The presence of Tregs is considered an important part of the immunosuppression associated with this malignancy. More recently, the cells of the TME of cHL have been characterized at the single cell level with respect to transcription and also location (4). With the growth of RS cells that exhibit low levels of MHC-II, tumors are populated by Lag3+ T cells. With the growth of RS cells that express high levels of MHC-II, other checkpoint inhibitors may be expressed on infiltrating T cells.

## Managing the TME – interventions to increase activity of anti-cancer agents including antibody-based agents

**Figure 3:**
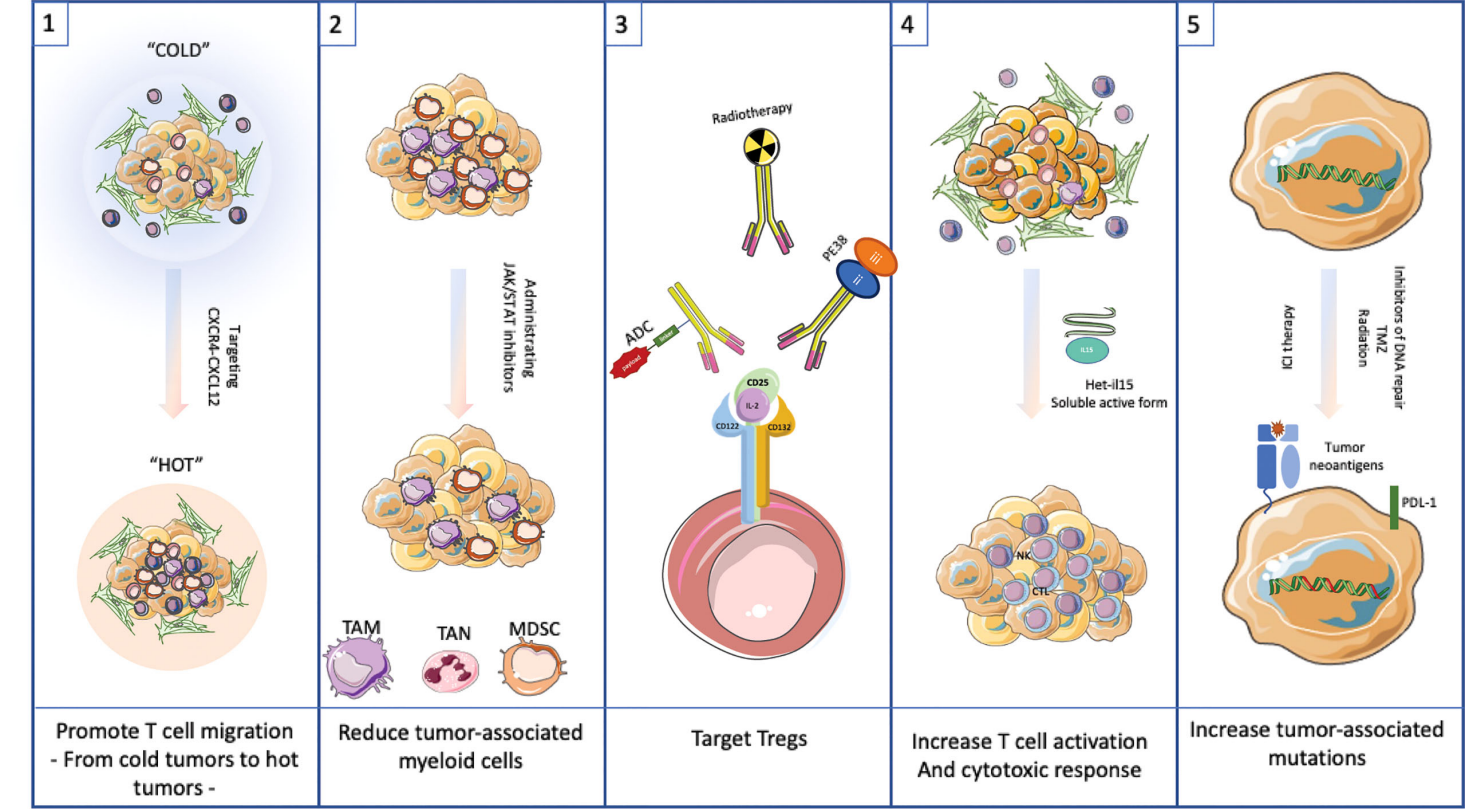
Targeting the TME as possible therapeutic approach.**(1)** Promote T cell migration into cold tumors by targeting CXCR4-CXCL12 interaction. **(2)** Reduce tumor-associated myeloid cells by using JAK/STAT inhibitors. **(3)** Target Tregs with antibodies to CD25 or similar surface antigen. **(4)** Increase T cell activation by adding Het-IL15. **(5)** Increase tumor-associated mutations by administrating inhibitors of DNA repair, TMZ or radiation to allow neoantigens to drive a robust immune response.

Possible approaches to managing the TME without undue risk to the cancer patient are discussed below and presented in **Figure 3**. Five individual strategies are discussed. These include promoting T cell migration into cold tumors, reducing myeloid populations, targeting Tregs, increasing the activity of T cells to promote a cytotoxic response and finally to generating additional tumor-associated mutations. Each approach has a potential downside that will need to be managed carefully. For each option below, we outline an approach to modify the TME. These options are unlikely to be curative. Rather they are treatments that could be combined with checkpoint inhibitors or even traditional chemotherapeutic agents to produce enhanced results over current therapies. Before describing management options it is important to emphasize the inter and intra-patient TME heterogeneity that will need to be assessed with molecular diagnostic tools before embarking on a specific therapeutic intervention (53, 54).

Finally, the management of the TME must strategies that promote efficient antigen presentation allowing anti-tumor T-cells to function. Minimally, this should include strategies to maintain or increase the expression of functional surface MHC-I antigens and the associated antigen presentation apparatus, possibly through the use of histone deacetylase inhibitors (55). Especially in PDAC, but possibly in other cancers, the loss of surface MHC-I is mediated by autophagy suggesting another route of pharmacological internvention (49).

### Option 1

Promote the migration of T cells into the TME. This strategy amounts to converting a cold tumor to a hot one. One approach, outlined by the Fearon Lab, proposes to break the restraining interactions between CXCR4 and CXCL12 which keeps T cells at the periphery of tumors (12, 13). Another approach is to recruit CD4 and CD8 cells into cold tumors using type 3 innate lymphoid cells (ILC3s) (56). The ILC3s secrete CXCL10 which is the recruiting signal for the attraction of CD4 and CD8 cells. Of course, there has be a protocol to recruit ILC3s into tumors or to produce intratumoral CXCL10 by synthetic means, perhaps even the use of mRNA transcripts injected directly into cold tumors.

### Option 2

Reduce myeloid accumulation and or myeloid-mediated suppression in the TME by interfering with the cytokine pathway of chemoattraction or activation. Agents like JAK, BTK, PI3K and TKI inhibitors have reported this kind of activity. Likewise, interfering antibodies that soak up activating cytokines could be useful to reduce myeloid cell suppression. Regarding the latter, Hailemichael et al. have described the use of IL-6 blockade to reduce immunotherapy related autoimmunity (i.e. immune related enterocolitis) through the use of anti-IL-6 antibodies. In their analysis they noted the high expression of IL-6 and other cytokines from intestinal tissues of patients treated with ICB and experiencing immune related enterocolitis. Because many of these cytokines signal *via* the JAK-STAT pathway, small molecular weight inhibitors might also be useful in blunting autoimmune toxicity (42). Bruton’s Tyrosine Kinase (BTK) is a well-known target expressed in certain B-cell malignancies including mantle cell lymphoma and chronic lymphocytic leukemia, and several BTK inhibitors are approved for human use. However, BTK is also expressed in cells of myeloid lineage and might be a therapeutic target in cells of the TME (40). Myeloid cells expressing BTK include monocytes, macrophages, thrombocytes, neutrophils and dendritic cells. The role of BTK in each of these cell types has not been clearly defined. However, various investigators have proposed using BTK inhibitors to alter the composition of the TME to a more favorable state for cancer treatment outcomes.

For example, MDSCs which are known to inhibit T cell function, express BTK. These investigators reported the use of the BTK inhibitor, ibrutinib, to reduce nitric oxide produce and slow cell migration. Further, the mRNA levels for indolamine 2,3 dioxygenase were reduced. Further, MDSCs in tumor and spleen of tumor-bearing mice were reduced in frequency when animals were treated with ibrutinib. A potential combination treatment was explored in tumor bearing mice where anti-PD-1 in combination with ibrutinib reduced tumor growth to a greater extent than either single agent treatment (57).

Reports using select PI3K inhibitors suggest potential use toward achieving similar results. Sun et al. report on the compound CYH33 (an inhibitor of the PI3K alpha isoform) (43). CYH33 exhibited a potent antitumor activity in an immune-competent context by enhancing the infiltration and activation of CD8+T and CD4+T cells, while attenuating M2-like macrophages and regulatory CD4+T cells. This adds to the literature on inhibitors of the PI3K delta isoform where Tregs are inhibited breaking immune tolerance.

Tyrosin Kinase inhibitors (TKIs) (such as Imatinib and Nilotinib) are used for the treatment of hematological neoplasms. Upon exposure to these inhibitors, large numbers of monocytes are killed, while NK cells are highly resistant and remain vialble. Further, TKIs can revert the M2 immunosuppressive polarization, favoring the M1-oriented macrophages facilitating antitumor immune responses *via* activation of NK cells (58).

### Option 3

Eliminate or reduce the population of Tregs. CD25 is a prominent surface marker on Tregs and has been the focus of targeting by antibodies or antibody-modified cytotoxic agents (59–61). The downside to the this approach is the risk of nullifying the functionality of activated T cells responding to IL2, although one report suggests that CD25 blockade does not interfere with vaccine driven T cell responses, so there is reason to believe that CD25 blockade or targeting with cytotoxics will permit CD8+ CTLs to function (62). Another possible approach is the intratumoral injection of cytotoxic agents targeting CD25, thus relieving the local suppressive activity without systemic risk to all T cell function (61).

### Option 4

Increase the activation of T cells. This is independent of checkpoint inhibitors. There are several iterations of IL15 that can be administered to activate T cell function and could be used to stimulate the antitumor action of T cells that remain ‘poor performers’ (63). Here a bioactive form of IL15, namely the heterodimer of IL15 and IL15 receptor alpha, is used to promote the infiltration and persistence of either endogenous or adoptive T cell populations. Following this theme, it may be possible to combine the properties of IL15 and ICB into a single bioactive agent. Knudson et al. describe a bi-functional agent that includes IL15 linked to anti-PD-1 antibody sequences (64). Their report suggested that this bifunctional agent enhanced intratumoral lymphocyte numbers including CD8+ T cells and NK cells. There was also a reduction in immunosuppressive and pro-tumorigenic immune cells in the TME, including Treg, M2-like macrophages and M-MDSC.

### Option 5

Increase mutational load within malignant cells. This is a risky approach but one likely to generate neoantigens that can be used by T cells to eliminate refractory tumor cells. Additional mutations can be introduced in a number of ways. DNA modifying agents such as temzolomide or other alkylating agents can introduce mutations *via* chemical modification. This approach suffers from the lack of tumor-specific targeting. Mutations will likely be introduced into normal cells as well as malignant cells and these may be the source of secondary cancers. Another approach is targeting DNA repair systems. We know that mismatch repair defective cancers respond very well to ICB, including a recent report suggesting a very high response rate when the defect included loss of key mismatch repair enzymes. When considering this general approach it will be important to establish the relative utility of repair inhibitors versus loss (silencing or deletion) of key components of the repair system. If it turns out that loss of expression of key components is necessary to get best results, this suggests the use of SI RNA or the CRISPR methodology where genes of key components are targeted for knockdown or knockout. If this could be achieved using selected disruption within malignant cells, this approach would provide a treatment to maximize ICB activity without involving the use of systemic mutagens. PARP inhibitors are particularly useful in treating patients with BRCA1/2 mutations where the accumulation of DNA damage leads to cell death. When this approach produces responses but is not curative, the addition of ICB might be useful. Support for this approach has been published (65).

## Conclusion

Here we outline approaches to manage or modify the TME with the goal of achieving better responses to therapeutic agents. While we mention specific strategies, the overall goal is to maximize responses with minimum additional risk to individual cancer patients. Not only are TME-modifying agents going to be important but their use in specific timelines are likely to be crucial. Therapeutic interventions, especially combination therapies, will be coordinated with precision whenever possible.

## Author contributions

MTB wrote the manuscript and created the figure and table. AA reviewed the manuscript. DF edited and reviewed the manuscript, the table and the figure. All authors contributed to the article and approved the submitted version.

## Funding

Authors are supported by internal funding from the Center for Cancer Research, National Cancer Institute, National Institutes of Health, DHHS.

## Conflict of interest

The authors declare that the research was conducted in the absence of any commercial or financial relationships that could be construed as a potential conflict of interest.

## Publisher’s note

All claims expressed in this article are solely those of the authors and do not necessarily represent those of their affiliated organizations, or those of the publisher, the editors and the reviewers. Any product that may be evaluated in this article, or claim that may be made by its manufacturer, is not guaranteed or endorsed by the publisher.

## Abbreviations

TME, tumor microenvironment; TAMs, tumor-associated macrophages; TANs, tumor associated neutrophils; NK, natural killer; DCs, dendritic cells; CTLs, cytotoxic T lymphocytes; Tregs, regulatory Tcells; CAFs, cancer-associated fibroblasts; ARG-1, arginase; ROS, reactive oxygen species; NO, nitric oxide; IL, interleukin; IFNs, interferons; TNF, Tumor necrosis factor; TGF, transforming growth factor; CCL, C-C motif chemokine ligand; CCR, C-C motif chemokine receptor; CXCL, C-X-C motif chemokine ligand; CXCR, C-X-C motif chemokine receptor; M-CSF, Macrophage colony-stimulating factor; SDF-1, stromal cell-derived factor 1; ICB, immune checkpoint blockade; PD-1, programmed cell death protein 1; PD-L1, PD-1 ligand; ECM, extracellular matrix; NETs, neutrophil extracellular traps; MHC, major histocompatibility complex; cHL, classic Hodgkin Lymphoma; PDAC, Pancreatic Ductal Adenocarcinoma; GBM, Glioblastoma Multiforme; TMZ, temozolomide.
